# Transcranial magnetic stimulation in forensic populations: scoping review

**DOI:** 10.3389/fpsyg.2026.1834732

**Published:** 2026-06-09

**Authors:** Ana Carolina Dias, Ana Rita da Cruz, Carolina Dall'Antonia da Motta

**Affiliations:** 1 School of Psychology and Life Sciences, Lusófona University, Lusófona University, Lisbon, Portugal; 2 Digital Human-Environment Interaction Lab, Lusófona University, Lisboa, Portugal; 3 Center for Research in Neuropsychology and Cognitive and Behavioral Intervention, University of Coimbra, Coimbra, Portugal

**Keywords:** antisocial behavior, criminal behavior, forensic population, scoping review, transcranial magneticstimulation (TMS)

## Abstract

**Introduction:**

The literature discusses the association between criminal behavior and brain functional dysfunctions, underscoring the relevance of investigating and intervening on the neural correlates and structures associated with criminal and antisocial behavior. In this context, transcranial magnetic stimulation (TMS) has been used to explore brain functioning and has shown significant effects in modulating cognitive and affective functions and as an intervention for various pathologies. This scoping review aims to explore how TMS has been used in forensic context, providing an overview of the TMS patterns used, the respective protocols, and the main conclusions drawn from these investigations, as well as their implications.

**Methods:**

The literature search was carried out on b-ON, EBSCO and PubMed, and included all studies carried out up to the date of the search, written in Portuguese, English, French and Spanish.

**Results:**

Three studies were identified for review, which demonstrate the potential of TMS in screening and diagnosing functional deficits associated with criminal behavior. It also highlights the untapped potential of TMS as an intervention with forensic populations, as well as the need for research with more diverse samples.

## 1 Introduction

Forensic populations are composed of individuals, young people or adults, that are involved with the justice system (Jones et al., 2019) because they adopted “any overt or covert law-breaking conduct in a given country or state” (Morizot and Kazemian, 2015). These behaviors are considered criminal and manifest as antisocial behaviors (e.g., impulsiveness, aggressiveness, etc.; Lugo, 2015). Research in this field indicates that the adoption of these behaviors results from the convergence of several factors, including developmental, environmental, biological, and psychological factors (Bartol and Bartol, 2017; Kamaluddin et al., 2015; Zabarniy et al., 2023). Additionally, the literature suggests that the criminal behavior is associated with deficits in moral processing (Bandura, 1999; Glannon, 2014; Kiriakidis, 2016), that is, with the “failure of self-regulation of learned standards of behavior that guide thinking, feeling, and acting” (Kiriakidis, 2016). According to Raine (2019), individuals who manifest antisocial behaviors present dysfunctions in at least one brain area related to moral processing. Neurologically, moral cognition depends on the interaction between multiple affective and cognitive systems, and its circuit involves cortical and subcortical structures (Glannon, 2014; Moll et al., 2008; Tuck and Glenn, 2021; Young and Dungan, 2012).

It is, therefore, relevant to investigate the structures associated with criminal and antisocial behavior, not only to understand their origins, but also to identify and develop potential interventions. For that purpose, the role of non-invasive cerebral stimulation techniques is highlighted, such as transcranial magnetic stimulation (TMS). TMS is a neuromodulation technique that consists on the manipulation of neuronal polarity and excitability in specific brain areas (Ling et al., 2020; Sun et al., 2023; Wieczorek et al., 2021; Yen et al., 2024). This phenomenon occurs through action potential induction, which results in the alteration of neurotransmitters release (such as glutamate and gamma-aminobutyric acid—GABA-, responsible for the activation and inhibition, respectively, of neuronal activity), promoting synaptic plasticity (Sun et al., 2023; Yen et al., 2024). This procedure is performed by positioning an electromagnetic impulse induction coil on the scalp, which interacts with the neurons to a depth of 4 cm (Ling et al., 2020; Oberman, 2014; Wieczorek et al., 2021).

TMS is prescribed by a licensed clinician with knowledge of brain physiology (e.g., master's in psychology, neurology, and neurosurgery, etc.), who decides which protocol to follow based on the patient needs, and administered by a technician (Feifel et al., 2018; Fried et al., 2021; Marder et al., 2022). Although no specific qualification is required for the technician, this role is often performed by health professionals (e.g., nurses, psychiatrists, etc.), and it is recommended that they have basic life support training (Feifel et al., 2018; Fried et al., 2021; Marder et al., 2022). The technician is supervised by the clinician, and has the most regular contact with patients, performing patient monitoring functions (Fried et al., 2021). Both the clinician and the technician must undergo rigorous and specific training related to TMS, including aspects such as handling the device, patient monitoring and side effects response, to ensure proper treatment (Lefaucheur et al., 2014; Marder et al., 2022).

TMS is considered a safe procedure, and there are no reports of permanent neurological, cognitive and cardiovascular sequels (George and Aston-Jones, 2010). Nonetheless, it has some side effects associated with it, whose manifestation and severity depends on the utilized TMS protocol (Fried et al., 2021; Klomjai et al., 2015). In this context, the most frequently manifested side effects are: migraines, localized discomfort (in the stimulated and adjacent areas) and temporary spasms in the eyebrow, brow, and/or chin (Fried et al., 2021; Marder et al., 2022). With lower frequency, are reported the occurrence of seizures (risk lower than 0.5%; George and Aston-Jones, 2010) and mood changes (specific to certain protocols and/or stimulated areas; Fried et al., 2021; Marder et al., 2022). Benninger et al. (2021) alert for the increased susceptibility to seizures in individuals with certain health problems or drug related problems. In parallel, TMS is a noisy procedure, which can cause a temporary increase in auditory threshold after administration, as such, the use of ear protection is recommended for everyone involved (George and Aston-Jones, 2010; Marder et al., 2022).

Different pulse patterns can be applied through this technique (i.e., single-pulse, paired-pulse and repetitive pulse), depending on the purpose of its use. Through single-pulse TMS (spTMS), isolated pulses are induced in a specific cortical area, with a fixed interval between them (e.g., 5 s; Chail et al., 2018; Farzan, 2014). This stimulation pattern is frequently combined with electrophysiological (e.g., electroencephalography—EEG—and electromyography—EMG) and neuroimaging (e.g., functional magnetic resonance imaging) monitoring techniques, in order to explore cortical reactions and map brain functions (e.g., motor threshold, cortical silent period, etc.; Farzan, 2014).

Paired-pulse TMS (ppTMS) paradigm entails the application, in the same or in different hemispheres (Klomjai et al., 2015; Vahabzadeh-Hagh, 2014), of two successive pulses: a conditioning stimulus (CS) followed by a test stimulus (TS), with a variable interstimulus interval (i.e., depending on the purpose of the stimulation; Rossini et al., 2015; Vahabzadeh-Hagh, 2014). Through ppTMS, it is possible to investigate neuronal interactions, exploring intracortical and interhemispheric inhibition and facilitation (Klomjai et al., 2015; Rossini et al., 2015; Vahabzadeh-Hagh, 2014).

Both spTMS and ppTMS have been used to explore the functioning of specific brain areas, without long-lasting effects (Klomjai et al., 2015; Rossini et al., 2015; Rotenberg et al., 2014). Literature suggests that these techniques can be useful tools in identifying differences in cortical functioning of healthy individuals and individuals with various disorders, with diagnosing and intervening implications (Benninger et al., 2021). For example, Kang et al. (2019) have identified deficits in the cortical excitability of individuals with obsessive-compulsive disorder (i.e., shorter cortical silent periods and reduced intracortical facilitation), associating this factor to the onset of the disease symptoms. Chandra et al. (2016) highlight the usefulness of spTMS in identifying early stages dementias with cholinergic deficits (e.g., Alzheimer's disease) and in differentiating them from dementias without cholinergic deficits (e.g., frontotemporal dementia), which benefits not only the diagnosis of this population, but also the selection of intervention. Most recently, Lissemore et al. (2022) discovered that, through cortical facilitation (along with other demographic and clinical factors), it is possible to predict the response of individuals with late-onset depression to pharmacotherapy.

On the other hand, with the purpose of modulating cortical functioning, repetitive TMS (rTMS) consists of administering pulse trains capable of inducing excitability or inhibition of a certain cortical area activity (Benninger et al., 2021; George and Aston-Jones, 2010; Klomjai et al., 2015; Lefaucheur et al., 2014; Oberman, 2014). Oberman (2014) clarifies that brain activity excitability is induced by applying repetitive trains of high-frequency pulses (higher or equal to 5Hz), with an interval of 20 to 30 s between them. The author also mentions that brain activity inhibition is induced by the continuous application of low-frequency pulses (lower or equal to 1Hz).

The rTMS paradigm has been used in the neuropsychological field to intervene in several pathologies, such as depression, type I bipolar disorder, schizophrenia (Wieczorek et al., 2021) and obsessive-compulsive disorder (Mantovani et al., 2010). It has also demonstrated significant effects for cognitive and affective functions modulation in community individuals, namely in the modulation of motor and temporal impulsivity (Yang et al., 2018b), of the ability to recognize emotional expressions (Balconi et al., 2011; Balconi and Canavesio, 2013, 2014), on the adoption of prosocial and empathic behaviors (Balconi and Canavesio, 2013, 2014; Yang et al., 2018a) and on the emotional processing of individuals with antisocial symptomatology (Konikkou et al., 2020). It should be emphasized that this stimulation pattern has been demonstrating long-lasting effects that transcend stimulation periods (Klomjai et al., 2015; Oberman, 2014).

The need for effective interventions that reduce the adoption of antisocial behaviors and prevent criminal behaviors is still pertinent. These interventions will, in turn, have an impact on reducing recidivism among individuals in the justice system. In this context, interventions aimed at modifying antisocial behavior have been implemented, such as individual and group therapies (e.g., focused on impulsiveness, interpersonal difficulties, etc.), behavioral modification programs (e.g., focused on reducing criminal perpetration, etc.) and pharmacological therapies, however, without much success (Gibbon et al., 2020; Glannon, 2014; National Institute of Health and Clinical Excellence [NICE], 2013; Tuck and Glenn, 2021). Therefore, the relevance of TMS investigation should be noted, not only as a method for investigating neural circuits, but also as an intervention method.

Considering the above, this scoping review intends to explore how TMS has been used in forensic settings. Therefore, it is aimed to: (a) identify the TMS patterns used with forensic population; (b) understand the differences between the used protocols; (c) provide an overview of the main conclusions reached through this neuromodulation technique; (d) identify gaps in research on the topic; (e) and discuss the utility of TMS in forensic settings.

## 2 Methods

This paper uses the methodology of scoping review, in order to explore and synthesize the existing literature (Anderson et al., 2008; Colquhoun et al., 2014) about the usage of TMS with forensic populations, following the guidelines and checklists of the extension for scoping reviews of the Preferred Reporting Items for Systematic Reviews and Meta-Analyses (PRISMA-ScR; Tricco et al., 2018). In order to access the articles quality, the Strengthening the Reporting of Observational Studies in Epidemiology initiative (STROBE; Vandenbroucke et al., 2014) and the Joanna Briggs Institute (JBI; Moola et al., 2020) checklists were also used.

### 2.1 Eligibility criteria

This review includes all scientific studies conducted up to the date of the research, written in Portuguese, English, French and Spanish, regardless of whether or not they were peer-reviewed (e.g., unpublished study reports). Literature review articles, meta-analyses, books, and ebooks were excluded.

### 2.2 Research strategy

The literature research was conducted through b-ON and EBSCO database aggregators (in which all databases were selected) and the PubMed database, combining search terms related to TMS and the forensic population (Table 1). The search included any article developed to date and in any language. A manual search of articles was also performed by analyzing the reference lists of the extracted articles (snowball technique for references that may not have been initially identified).

**Table 1:** Searching equations.

Aggregator/data base	Searching equations
b-ON	This searching equation was used in both the b-ON and EBSCO aggregators.
EBSCO	
PubMed	(“transcranial magnetic stimulation“) AND (inmates). (“transcranial magnetic stimulation“) AND (prisoners). (“transcranial magnetic stimulation“) AND (offenders). (“transcranial magnetic stimulation“) AND (forensic population).

### 2.3 Selection strategy

The records resulting from the search were imported into the Rayyan QCRI (Ouzzani et al., 2016) software, in order to expedite the screening of eligible studies for review. Firstly, duplicated records were identified and removed, as well as records written in an ineligible language. Then, the title and abstracts of the articles were analyzed, and those that did not align with the study's goals were eliminated. This process, which involved the collaboration of a second researcher to screen 4% of the abstracts, resulted in the identification of records for full-text reading and inclusion in the review.

### 2.4 Data extraction strategy

The information extracted from the articles corresponds to its year and journal of publication, country of origin of the study, goals, sample characteristics, stimulation pattern (i.e., spTMS, ppTMS or rTMS), measure used, stimulated brain area, utilized devices and main results. The data organization was performed with the support of Microsoft Excel software (version 2501).

## 3 Results

**Figure 1:**
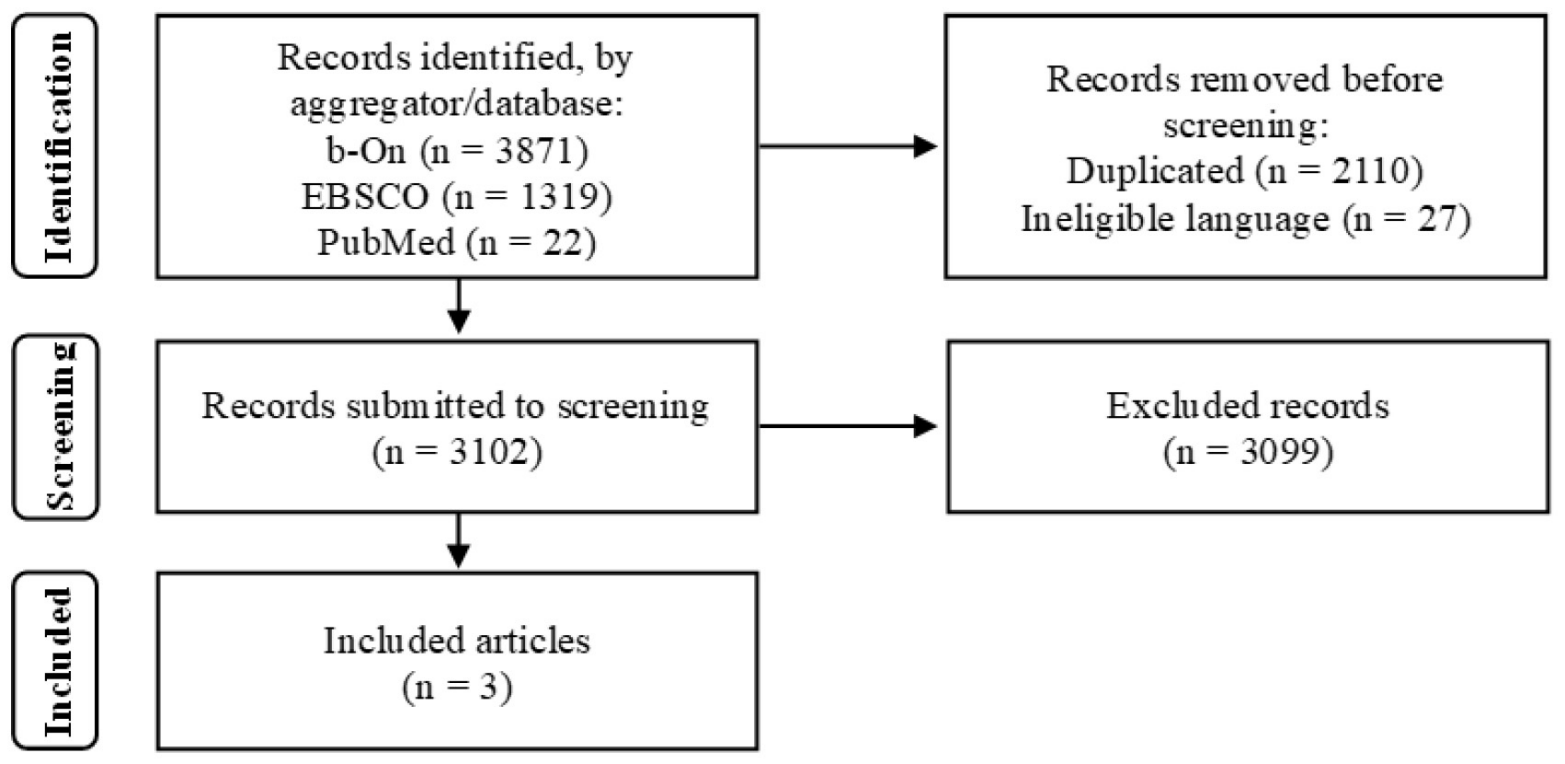
Flow of records.

The literature search yielded 5,212 records. After identifying and removing duplicated records and records written in an ineligible language, the title and abstracts of the remaining articles were analyzed. This process culminated in the selection of three articles for full reading and its integration in the revision. The flow of records is depicted in Figure 1.

Bibliographic characteristics of the included articles are summarized in Table 2. The included studies were published in English, between the years of 2011 and 2014, and are associated with Canadian and German institutes/research groups. According to SCImago Journal and Country Rank, the scientific journals in which these articles were published are highly regarded in the psychology and neuropsychology field, ranking in the top quartile of the classification table for the year of 2023. Additionally, the studies follow the STROBE overall recommendations for reporting research (Vandenbroucke et al., 2014; see Appendix A), and show methodological quality (Moola et al., 2020; see Appendix B).

**Table 2:** Bibliographic characteristics.

Authors	Year	Country	Journal
Hoppenbrouwers et al.	(2012)	Canada	*Cortex* (SJR = 1.33; Q1).
Hoppenbrouwers et al.	(2014)	Canada	*Journal of Psychiatry and Neuroscience* (SJR = 1.32; Q1).
Philipp-Wiegmann et al.	(2011)	Germany	*Neuropsychobiology* (SJR = 0.89; Q1).

SJR, SCImago Journal Rank; Q, journal quartile.

Indicators according to SCImago Journal and Country Rank, for the year of 2023.

Two of the studies have the goal to investigate intracortical inhibition (Hoppenbrouwers et al., 2012; Philipp-Wiegmann et al., 2011), whereas the third intends to explore interhemispheric connectivity (Hoppenbrouwers et al., 2014).

Table 3 provides a general description of the articles included in the revision, including its goals, sample and main results. The TMS protocols utilized are outlined in Table 4.

**Table 3:** General description.

Reference	Goals	Sample	Main results
Hoppenbrouwers et al. (2012)	Access cortical inhibition and excitability and working memory performance, in psychopathic offenders.	15 healthy individuals (*M* = 34 years); 13 psychopathic offenders (*M* = 34.2 years); right-handed; male.	Deficits in offenders' DLPFC inhibition, but not in MC; deficits in offenders' working memory performance; positive correlation between working memory performance and DLPFC intracortical inhibition in control group.
Hoppenbrouwers et al. (2014)	Explore functional interhemispheric connectivity, in psychopathic offenders.	15 healthy individuals (M = 32.1 years); 18 psychopathic offenders (*M* = 33.4 years); right-handed; male.	Increased signal propagation from right hemisphere to left hemisphere in offenders; cortical silent periods significantly longer in offenders' right hemisphere; no differences in intracortical short interval inhibition and intracortical facilitation; no correlations between PCL-R factors and TMS measures.
Philipp-Wiegmann et al. (2011)	Investigate primary MC inhibitory control, in violent offenders.	30 individuals with no history of violence and no criminal record (*M* = 30.4 years); 32 offenders convicted for violent crimes (*M* = 35.5 years); right-handed; male.	Reduced cortical inhibition in offenders; similar cortical facilitation between groups; no correlation between severity of violence and cortical inhibition/facilitation.

DLPFC, dorsolateral prefrontal cortex; M, mean; MC, motor cortex; PCL-R, Hare Psychopathy Checklist-Revised; TMS, transcranial magnetic stimulation.

**Table 4:** Protocols.

Reference	Pattern	Measure	Interstimulus Interval	Intensity	Brain Area	Stimulator	Coil
Hoppenbrouwers et al. (2012)	ppTMS	Long interval intracortical inhibition.	100ms	CS and TS > RMT.	MC and DLPFC; left hemisphere	2 stimulators Magstim 200; 1 module BiStim.	Standard coil; 7 cm of diameter; 45° of the midline sagittal line; arm pointing backward.
Hoppenbrouwers et al. (2014)	ppTMS.	Short interval intracortical inhibition.	2 ms and 4 ms.	CS 80% of RMT; TS > RMT.	MC; both hemispheres.	2 stimulators Magstim 200.	Standard coil; 7 cm of diameter; 45° of the midline sagittal line; arm pointing backward.
		Intracortical facilitation.	10 ms, 15 ms and 20 ms.	CS 80% of RMT; TS> RMT.	MC; both hemispheres.	2 stimulators Magstim 200.	Standard coil; 7 cm of diameter; 45° of the midline sagittal line; arm pointing backward.
		Interhemispheric inhibition.	10 ms.	–	MC; both hemispheres.	2 stimulators Magstim 200.	Standard coil; 7 cm of diameter e 8 cm de diameter.
	spTMS.	Interhemispheric signal propagation.	500 ms.	Impulse > RMT.	MC and DLPFC; both hemispheres.	2 stimulators Magstim 200; 1 module BiStim.	Standard coil; 7 cm of diameter; 45° of the midline sagittal line; arm pointing backward.
		Cortical silent period.	–	Impulse 140% of RMT.	MC; both hemispheres.	2 stimulators Magstim 200.	Standard coil; 7 cm of diameter; 45° of the midline sagittal line; arm pointing backward.
Philipp-Wiegmann et al. (2011)	ppTMS.	Short interval intracortical inhibition.	1 ms, 3 ms and 5 ms.	CS 80% of RMT; TS > RMT.	MC; both hemispheres.	1 stimulator MagPro X100; 1 module MagOption.	Standard coil; 6.5 cm of diameter; 45° of the midline sagittal line; arm pointing backward.
		Short interval intracortical facilitation.	7 ms, 9 ms, 11ms, 13 ms and 15 ms.	–	MC; both hemispheres.	1 stimulator MagPro X100; 1 module MagOption.	Standard coil; 6.5 cm of diameter; 45° of the midline sagittal line; arm pointing backward.

CS, conditioning stimulus; DLPFC, dorsolateral prefrontal cortex; MC, motor cortex; ppTMS, paired-pulse transcranial magnetic stimulation; RMS, resting motor threshold; spTMS, single-pulse transcranial magnetic stimulation; TS, test stimulus.

### 3.1 Sample

The included studies in this scoping review resort to samples composed of adult individuals (i.e., aged 18 years or older), male, and right-handed (identifying the right hand as dominant).

Hoppenbrouwers et al. (2012) sample is composed of 15 healthy individuals and 13 psychopathic offenders (i.e., individuals who scored 25 or higher in Hare Psychopathy Checklist-Revised 2—PCL-R:2), the latter being recruited in a mental health program and transition houses. In Hoppenbrouwers et al. (2014) investigation 15 healthy individuals were recruited by self-selection through advertisements and 18 psychopathic offenders in a mental health program and transition houses. In both investigations, the exclusion criteria were the same: individuals under 18 and over 65 years of age; diagnoses of schizophrenia, schizophreniform/psychotic, bipolar, affective, or anxiety disorders, or any comorbid personality disorder; and history of substance abuse or dependence in the 6 months prior to the study.

Philipp-Wiegmann et al. (2011) resorted to 30 individuals with no history of violence and no criminal record, and 32 offenders convicted for violent crimes, referred by legal authorities. The exclusion criteria were insufficient German language skills, inability to read, intellectual disabilities, low intelligence (IQ < 85), a DSM-IV axis I diagnosis, and history of neurological events (e.g., brain injury or any type of vascular, inflammatory, or degenerative brain disorder). Participants also had to have a negative toxicology screen, and for the offender group, only individuals with frequent aggressive and violent behaviors were included.

Two of the investigations (Hoppenbrouwers et al., 2012, 2014) mention having submitted the participants to a clinical interview, in order to identify TMS contraindications. None of the studies mention having done a post-stimulation assessment to identify side effects or the maintenance of positive effects.

### 3.2 Stimulation pattern

Only one study (Hoppenbrouwers et al., 2014) indicates the number of sessions conducted (two), as well as the duration of each session (16-h TMS and EEG session, and 12-h TMS single session).

The use of paired-pulse (ppTMS) and single-pulse (spTMS) stimulation patterns was identified, with ppTMS being the most widely used technique.

#### 3.2.1 Paired-pulse TMS (ppTMS)

Cortical excitability is often assessed by ppTMS, through measures such as intracortical inhibition (including short interval and long interval), intracortical facilitation (including short interval) and interhemispheric inhibition and facilitation (Chen et al., 2022; Vahabzadeh-Hagh, 2014). Cortical inhibition is primarily mediated by GABA neurotransmitters, which allows the modulation of cortical excitability and neuroplasticity (Daskalakis et al., 2007; Ziemann et al., 2015), whereas cortical facilitation is strongly influenced by glutamate, which increases neuronal excitability and responsiveness to subsequent stimuli (Ziemann et al., 2015). Interhemispheric inhibition and facilitation reflect the suppression and potentiation, respectively, of cortical excitability between homologous cortical areas across hemispheres, via transcallosal pathways (Meyer et al., 1995).

Only one study used short interval cortical inhibition (Hoppenbrouwers et al., 2012), in which were induced supralimiar CS followed by supralimiar TS, with an interval of 100 ms between them. According to the literature, in this paradigm, CS should be induced at an intensity of 100–130% of resting motor threshold (RMT), and TS at 120%, with an interval of 50 to 200 ms (Chen et al., 2022; Vahabzadeh-Hagh, 2014). Hoppenbrouwers et al. (2014) e Philipp-Wiegmann et al. (2011) and their respective collaborators explored short interval cortical inhibition. Both defined CS intensity at 80% of RMT and TS above it, which is in line with the literature: CS at about 50% to 90% of RMT and supralimiar TS (Chen et al., 2022; Vahabzadeh-Hagh, 2014). The interstimulus interval used was also the recommended (i.e., 1 to 6 ms; Chen et al., 2022; Vahabzadeh-Hagh, 2014).

To investigate intracortical facilitation, Hoppenbrouwers et al. (2014) used interstimulus intervals between 10 and 20 ms (as recommended: 8–30 ms; Chen et al., 2022; Vahabzadeh-Hagh, 2014), and the same intensities defined for inhibition: sublimiar CS and supralimiar TS (Chen et al., 2022; Vahabzadeh-Hagh, 2014). Philipp-Wiegmann et al. (2011) are not clear about the used pulse intensity to investigate short interval intracortical facilitation, however, it is recommended the induction of CS at 100–130% of RMT and TS at 90% of RMT (Chen et al., 2022; Vahabzadeh-Hagh, 2014). The interstimulus interval used was between 7 and 15 ms, which exceeds what is proposed by the literature (i.e., 1.0–1.5 ms, 2.3–3.0 ms, 4.1–5.0 ms; Chen et al., 2022; Vahabzadeh-Hagh, 2014).

One of the studies also analyzes interhemispheric inhibition (Hoppenbrouwers et al., 2014). The authors do not clarify the pulse intensity. Nevertheless, considering the interstimulus interval presented (10 ms), it can be inferred that suprathreshold stimuli were used (Chen et al., 2022; Vahabzadeh-Hagh, 2014).

#### 3.2.2 Single-pulse TMS (spTMS)

Only one study uses spTMS (Hoppenbrouwers et al., 2014), to explore interhemispheric signal propagation and cortical silent period. Interhemispheric signal propagation assesses evoked cortical activity transmission between the stimulated hemisphere and the contralateral hemisphere (Voineskos et al., 2010). The analyzed timeframe corresponds to what is frequently used in other investigations: 50–150 ms for the stimulated hemisphere and 60–160 ms for the contralateral hemisphere (e.g., Hui et al., 2020, 2021; Voineskos et al., 2010). One hundred pulses were administered (similar to other studies, e.g., Hui et al., 2020, 2021; Voineskos et al., 2010), with an interval of 5 s between them (varying intervals are presented in the literature; e.g., Casula et al., 2020; Tecchio et al., 2023; Voineskos et al., 2010). It used a supralimiar stimulus intensity.

The cortical silent period corresponds to the period in which EMG activity is temporarily interrupted after the induction of a motor evoked potential during voluntary muscle contraction (in this case, the *abductor pollicis brevis* muscle; Fuhr et al., 1991). In order to investigate this measure, Hoppenbrouwers et al. (2014) administered 10 pulses with an intensity of 140% of RMT, as recommended in literature (Chen et al., 2022; Farzan, 2014). Chen et al. (2022) emphasize that EMG should record the period between 100 ms before TMS and 400 ms after TMS, given that the duration of the cortical silent period is approximately 200–300 ms. These data are not disclosed by Hoppenbrouwers et al. (2014), and it is also unclear the interstimulus interval used.

### 3.3 Stimulated brain area

Hoppenbrouwers et al. (2014) only investigate the functioning of the left hemisphere, whereas the remaining studies focus on both hemispheres. The motor cortex (MC) stands out as a target of stimulation present in all studies, as has been widely reported in the literature (e.g., Chen et al., 2022; Klomjai et al., 2015). In this context, various muscles from which motor evoked potentials are recorded can be used, including the *abductor pollicis brevis* muscle and the first *dorsal interosseus* muscle (as used by the authors), among others (Vahabzadeh-Hagh, 2014).

Two of the studies also investigate the dorsolateral prefrontal cortex (DLPFC; Hoppenbrouwers et al., 2012, 2014). It should be noted that, in Hoppenbrouwers et al. (2014) study, the DLPFC is stimulated specifically to explore interhemispheric signal propagation.

### 3.4 Device used

The device used for TMS is, generally, composed by a stimulator and one or more coils (Rotenberg et al., 2014). Modules can also be used, to increase the device's efficiency/functionalities.

#### 3.4.1 Stimulator

Two of the studies (Hoppenbrouwers et al., 2012, 2014) use two units of MagStim 200. On its own, this stimulator is suitable for administering single monophasic pulses (spTMS), with a frequency of 0.5Hz (Magstim, 2024). By adding a BiStim module to connect two units, as seen in these two studies, the equipment extends its capacity to deliver not only single-pulses but also monophasic paired-pulses (ppTMS; Magstim, 2024). However, the maximum frequency drops to 0.25Hz (Magstim, 2024).

On the other hand, Philipp-Wiegmann et al. (2011) work with the MagPro X100 stimulator, with a MagOption module. This equipment is capable of inducing any stimulation pattern (spTMS, ppTMS and rTMS), through monophasic and biphasic pulses, with a frequency of up to 100Hz (Tonica Elektronik A/S, 2010).

#### 3.4.2 Coil

The coil used for stimulation (circular coil, H-coil or standard coil) typically varies depending on the purpose of the stimulation. That is, different types of coils can generate different magnetic field patterns, as well as more or less focused stimulation (Chen et al., 2022; Epstein, 2008; Rotenberg et al., 2014). The type of coil most commonly used in research and clinically is the standard coil (also known as figure-of-8) with a diameter of 7 cm (Chen et al., 2022; Epstein, 2008).

All studies analyzed administer stimulation through standard coils, two of which (Hoppenbrouwers et al., 2012, 2014) use coils with a diameter of 7 cm, and one (Philipp-Wiegmann et al., 2011) uses coils with a diameter of 6.5 cm. Additionally, Hoppenbrouwers et al. (2014) use an 8 cm diameter coil to stimulate the right hemisphere when studying interhemispheric inhibition. In line with the literature (Chen et al., 2022), all three investigations keep the coils under the cortex at a 45-degree angle from the median sagittal line, with the arm pointing backward.

### 3.5 Main results

Hoppenbrouwers et al. (2012) found significant differences in cortical inhibition between the control group and the experimental group (*F*_2, 25_ = 4.807; *p* = 0.017; $\eta_{\text{p}}^{2}$ = 0.278). Specifically, compared to the control group, offenders showed deficits in DLPFC inhibition (*F*_1, 26_ = 9.229; *p* = 0.005; $\eta_{\text{p}}^{2}$ = 0.262), but not in MC inhibition (*F*_1, 26_ = 2.238; *p* = 0.147; $\eta_{\text{p}}^{2}$ = 0.079). The offenders group demonstrated deficits in working memory, as evidenced by significantly lower performance on the Letter-Number Sequence test of the Weschler Adult Intelligence Scale III (WAIS-III; *t*_(26)_ = −3.063; *p* = 0.005). In this regard, there was a positive correlation between working memory performance and intracortical inhibition in the DLPFC of the control group (*r* = 0.679; *p* = 0.005). Although not statistically significant, this trend was also observed in offenders (*r* = 0.519; *p* = 0.069).

Hoppenbrouwers et al. (2014) identified significant differences in interhemispheric signal propagation between the control group and the experimental group (*F*_1, 25_ = 5.375; *p* = 0.029; $\eta_{\text{p}}^{2}$ = 0.177). These differences translate into a deficient connectivity between the hemispheres of psychopathic offenders, specifically through greater signal propagation from right to left hemisphere. Anomalies were also identified in the intracortical inhibition of the right hemisphere of offenders, with significantly longer periods of cortical silence (*F*_1, 26_ = 4.267; *p* = 0.048).

In the study by Philipp-Wiegmann et al. (2011), with an interstimulus interval of 3 ms, offenders had significantly higher motor evoked potential ratios than the control group (Z = −2.310; *p* = 0.021). However, no significant differences in overall cortical inhibition and facilitation were identified between the two groups (control: *F*_(60)_ = 1.183; *p* = 0.281; experimental: *F*_(60)_ = 0.016; *p* = 0.899). To control for the effects of violence severity on cortical inhibition and facilitation, the offender group was divided into four subgroups: homicide, manslaughter, bodily harm, and serious bodily harm. No statistically significant differences were found between the groups.

## 4 Discussion

This scoping review intends to understand how TMS has been used in forensic context, identifying gaps in the literature and potential directions for future studies. In this context, we identified three studies that explore brain function using TMS in forensic samples.

The small number of studies identified may be due to several factors, including the stimulation equipment itself. That is, in addition to being an expensive device, it is also large (75.000 cm3 and about 90 kg; Valero-Cabré et al., 2017), limiting its portability and the possibility of sharing it among institutions. Additionally, it is worth noting the limited range of the stimulation, which occurs only up to 4 cm deep (Oberman, 2014; Wieczorek et al., 2021), limits its modulation for relatively superficial cortical and subcortical areas. This factor may influence the selection of TMS as a neuronavigation and neuromodulation technique.

The absence of recent studies (less than 10 years old) may be a consequence of these factors. However, the relevance given to research in a forensic context compared to, for example, research in a clinical context should be reflected. That is, there is a greater abundance of research using TMS in clinical context, not only recent, but also in general (e.g., Hernández-Sauret et al., 2024; Li et al., 2021). This phenomenon reflects the scarcity of investment in research with forensic populations.

All studies were published in highly prestigious journals, both currently and at the time of publication of each article. However, this should be interpreted with caution: a journal's impact factor reflects the average frequency with which published articles have been cited (Garfield, 1972). As such, it does not correspond to a measure of the scientific quality of the study. Nevertheless, it is possible to verify that, in general, the studies follow the STROBE recommendations (Vandenbroucke et al., 2014; see Appendix A), which contributes to the quality of the article in terms of reporting. From a methodological point of view, the studies present a good quality, according to the JBI critical appraisal tool (Moola et al., 2020; see Appendix B).

Bibliometric analyses indicate that the countries that contributed most to knowledge about TMS through the publication of articles, between 2008 and 2018, were the United States of America, Germany, Italy, the United Kingdom, and Canada (McLean, 2019; Zheng et al., 2020). This phenomenon may have contributed to the fact that the studies identified in this review were conducted in Germany and Canada. Furthermore, from an historical point of view, in addition to the United Kingdom, where the concept of TMS first emerged (Barker et al., 1985), Germany and Canada were two of the countries where TMS took center stage: Germany, in a research context, since the 1990s (George, 2024), and Canada, in a clinical application context, since 2002 (Downar et al., 2016).

The selected studies use restricted samples, composed of adult, male, right-handed individuals. The selection of these samples may be due to a predominance of males in the forensic population, not only in the juvenile justice system (e.g., Direção Geral de Reinserção e Serviços Prisionais [DGRSP], 2024; Puzzanchera et al., 2022), but also among adult offenders (e.g., Carson and Kluckow, 2023; Eurostat, 2024). Simultaneously, the majority of the population presents the right hand as dominant (Papadatou-Pastou et al., 2020). Nevertheless, the exclusion of female samples, different age groups (including children and juveniles or older adults), and different hand dominance restricts the generalization of the results (due to possible functional and structural variations), as well as the understanding of the impact of TMS on different forensic subgroups. It should be noted that TMS is a safe procedure for any age group (Allen et al., 2017; George and Aston-Jones, 2010), with children experiencing similar side effects to adults (Allen et al., 2017).

The studies analyzed used ppTMS and spTMS. The selection of the stimulation pattern to be used depends on the goal of the study: to map brain functions (spTMS) or to explore neuronal interactions (ppTMS). That said, both stimulation patterns demonstrate potential for investigating brain functioning and identifying functional deficits, under normal and pathological conditions. The stimulation protocols used (e.g., interstimulus interval, intensity, coil) in the different studies are relatively homogeneous with those indicated in the literature, with only slight variations and omissions. However, standardization of procedures is necessary to ensure the possibility of studies replication. It should also be mentioned that these protocols were developed based on research with healthy participants from the community or clinical populations (for review, see Farzan, 2014; Oberman, 2014; Vahabzadeh-Hagh, 2014), which may limit the ecological validity of the results in forensic contexts.

Still in the context of stimulation patterns, there is a lack of research using rTMS. The literature has demonstrated the potential of rTMS as an intervention method for various pathologies (Mantovani et al., 2010; Wieczorek et al., 2021) and in the neuromodulation of cognitive and affective functions in community samples (Balconi et al., 2011; Balconi and Canavesio, 2013, 2014; Konikkou et al., 2020; Yang et al., 2018a,b). However, its use approved by the Food and Drug Administration is still restricted to the treatment of depression, obsessive-compulsive disorder, and smoking cessation (Cotovio et al., 2023; Marder et al., 2022). Table 5 provides a summary description of its protocols (Cotovio et al., 2023; Marder et al., 2022).

**Table 5:** rTMS protocols approved by the food and drug administration.

Disorder	Brain area	Impulse	Intensity, Frequency	Sessions	Devices	Coil
Major depressive disorder.	Left DLPFC.	75 sets of 40 (4 s/each); interval between sets: 26 s.	120% of RMT; 10Hz.	1/day (20–30 days).	NeuroStar, Magstim, Magventure, Brainsway.	standard, H-coil.
Obsessive-compulsive disorder.	ACC, medial PFC.	50 sets of 40 (2 s/each); interval between sets: 20 s.	100% of RMT; 20Hz.	1/day (29 days).	Magventure, Brainsway.	standard, H-coil.
Smoking cessation.	PFC, insula.	60 sets of 30 (3 s/each); interval between sets: 15 s.	120% of RMT; 10Hz	1/day (15 days); 1/week (3 weeks).	Brainsway.	H-coil.

ACC, anterior cingulate cortex; DLPFC, dorsolateral prefrontal cortex; PFC, prefrontal cortex; RMT, resting motor threshold.

Several brain regions have been studied using TMS, namely areas in the motor, visual and prefrontal cortices, in the frontal and parietal lobes, and the cerebellum (Sharbafshaaer et al., 2024; Siebner et al., 2022). The brain areas stimulated in the investigations correspond to the MC and DLPFC. The MC has been portrayed in the literature as the main brain area investigated through TMS (e.g., Chen et al., 2022; Klomjai et al., 2015; Paus, 2008; Siebner et al., 2022). This may be due to its ease of access, serving as a reference point for locating other brain areas (Paus, 2008). Additionally, motor evoked potentials function as a physiological biomarker, allowing the intensity of the stimulus to be adjusted for each user (Siebner et al., 2022). At the same time, the DLPFC is involved in functions such as impulse regulation and self-control (e.g., Hare et al., 2009), decision-making (e.g., Sela et al., 2012), moral processing (e.g., Greene et al., 2004), and reward processing (e.g., Staudinger et al., 2011). Research into these processes (and deficits therein) may be useful in the study of criminal and antisocial behavior.

Regarding the equipment used, the MagPro X100 stimulator appears to be the most versatile for research purposes, allowing the administration of multiple stimulation patterns. As mentioned previously, the standard coil is the most widely used in research context, which is reflected in its use in the three studies reviewed.

Given the reduced number of articles identified and the methodological variations between them, it is difficult to achieve the objective of providing an overview of the main conclusions reached through TMS. Nevertheless, the studies here reviewed generally point to deficits in interhemispheric processing and intracortical inhibition in offenders. Deficient interhemispheric processing means that one of the hemispheres (in this case, the left) does not properly process information from the other hemisphere (Hoppenbrouwers et al., 2014). This phenomenon is associated with the adoption of antisocial behaviors (Hecht, 2011), just as good interhemispheric connectivity is associated with the adoption of prosocial behaviors (Ishihara et al., 2023). Additionally, the literature suggests that the right hemisphere is associated with the adoption of prosocial behaviors (e.g., self-control, compliance with social norms, etc.), while the left hemisphere is associated with antisocial behaviors (e.g., impulsiveness, aggressiveness, etc.; Hecht, 2011, 2014). Considering the above, this deficient connection may imply the impairment of functions such as the inhibition of antisocial behaviors. The deficits in intracortical inhibition identified reflect dysfunctions in the frontal lobe (Hoppenbrouwers et al., 2012; Philipp-Wiegmann et al., 2011), and are commonly observed in individuals who exhibit antisocial behaviors, particularly in the prefrontal cortex (Ling et al., 2019; Lugo, 2015; Yang and Raine, 2009). These translate into emotional deficits and difficulties in impulse and behavior control (Hoppenbrouwers et al., 2012; Philipp-Wiegmann et al., 2011; Yang and Raine, 2009) and, consequently, can lead to the adoption of criminal behaviors.

These findings are relevant, not only for understanding part of the etiology of criminal and antisocial behavior (recognizing that it depends on the influence of several factors), but also for intervention. Some of the methods that have been used in this regard are individual and group therapies (based, for example, on the cognitive-behavioral model, focusing on changing patterns of thinking and behavior; Morgan, 2019), and behavioral modification and skills acquisition programs (Gibbon et al., 2020; National Institute of Health and Clinical Excellence [NICE], 2013). However, potential difficulties that professionals may encounter are highlighted, particularly in establishing the therapeutic alliance, treatment gain maintenance through time and the client's commitment to the intervention process.

The use of pharmaceuticals has also been explored (e.g., selective serotonin reuptake inhibitors, used in the treatment of depression and anxiety, with effects on increasing aversion to causing harm to others; Levy et al., 2014), psychotropic drugs (e.g., MDMA, with effects on elements of social cognition; Macpherson et al., 2019), and hormones (e.g., vasopressin, with effects on prosocial behavior; oxytocin, with effects on morally significant behaviors; Tuck and Glenn, 2021). However, their effects are limited to the time during which the individual is under their influence (i.e., no long-term effects), which would imply long-term use (Macpherson et al., 2019). Since substance abuse (i.e., alcohol and drugs) is one of the most frequent comorbidities with antisocial behavior, it should be noted that routine administration of drugs as treatment for antisocial behavior may have an adverse effect in terms of substance abuse or negative drug-substance interactions (American Psychiatric Association [APA], 2013; National Institute of Health and Clinical Excellence [NICE], 2013). Simultaneously, these interventions affect the body in a non-discriminatory manner (i.e., not only in the area of focus of the intervention itself), which can have negative effects (e.g., increasing one characteristic at the expense of decreasing another; Levy et al., 2014). Therefore, the potential of rTMS as a method of modulating the aforementioned functional deficits is highlighted. As an example, Konikkou et al. (2020) demonstrated significant effects of rTMS of the DLPFC on emotional processing in individuals with antisocial symptomatology, increasing their ability to recognize emotional expressions. Furthermore, rTMS has been shown to be a procedure more focused on the target area of intervention, compared to the use of pharmaceuticals (Glannon, 2014).

It should also be noted that two of the studies used samples of psychopathic offenders, while the third consisted of violent offenders, without assessing the presence of psychopathy. According to the literature, individuals with psychopathy may present alterations in their brain structure or connections (cortical—e.g., ventromedial prefrontal cortex and insular cortex—and subcortical—e.g., amygdala and striatum; De Brito et al., 2021), which may have implications for brain functioning in offenders with these traits, as well as their response to stimulation, influencing the results/potential gains of this intervention. This phenomenon reflects the need for further research with this population, to understand the impact of psychopathy and other factors on brain functioning.

## 5 Limitations

One of the eligibility criteria for this review is the language of the studies, which must be written in Portuguese, English, French or Spanish. The exclusion of other languages may have influenced the number of records identified. For example, considering that Germany and Italy are among the countries that have published the most articles on TMS (McLean, 2019; Zheng et al., 2020), research written in their languages may have been discarded.

Another limitation that should be mentioned is the fact that the title of the review was not registered, which may lead to the existence of duplicate works. Furthermore, only 4% of the abstracts were screened by a second reviewer.

## 6 Conclusions

To date, this is the first scoping review to explore the use of TMS in forensic context. This review provides an overview of the TMS patterns used with forensic population and their respective protocols, as well as the main conclusions drawn from these investigations. It also offers insight into the implications of these issues in the forensic context.

With this in mind, this work highlights the scarcity of research exploring brain function through TMS in forensic populations, and emphasizes the potential of this technique in screening and diagnosing functional deficits that may be associated with criminal behavior, specifically through spTMS and ppTMS. Although no studies exploring rTMS in this population have been identified, its role in the clinical context, as well as its long-term effects, is noteworthy. In view of the above, it would be beneficial to invest in research into this technique as an intervention in the modulation of brain circuits associated with the adoption of criminal and antisocial behaviors.

Future studies should focus on the specificities of the forensic population, and include more representative samples (e.g., women, children/juveniles, prison population, etc.). Furthermore, the inclusion of individuals with and without psychopathy in investigations may prove beneficial in identifying functional differences and understanding their brain functioning.

One of the concerns that may arise from the use of TMS in forensic populations is the ability of individuals to consent to the procedure. In this context, Portuguese law safeguards this issue: “people in need of mental health care have the right not to be subjected to (...) transcranial magnetic stimulation without their written consent, except under the terms provided for in this law” (art.° 8.°, Law n.° 35/2023, Assembleia da República, 2023), namely, “when these techniques are medically prescribed, prove to be the best therapeutic alternative and the prescription is confirmed by two psychiatrists in addition to the prescribing physician” (art.° 12°, Law n.° 35/2023, Assembleia da República, 2023).

Criminal behavior can have effects at the individual level (e.g., physical harm to an individual), community level (e.g., perception of fear in the community), institutional level (e.g., financial harm to a company), and social level (e.g., expenditure of public money on imprisonment; D'Almeida and Fernández-Pacheco, 2015; Office for National Statistics, 2022). Quoting (Slutkin 2017):

“*Just as we know that it is more effective and cost-efficient to treat drug addiction as a health issue than to punish those addicted, likewise, it makes more sense to prevent violent events and provide treatment through methods that change behaviours and norms.”*

That said, research into the neural structures involved in the adoption of criminal and antisocial behaviors is essential, not only to understand its etiology, but also to plan effective interventions. Reflecting on the potential of TMS, a neuromodulation intervention using this technique, accompanied by individual therapy and/or integration into intervention programs (e.g., Moral and Ethical Development Program, Personal and Emotional Skills Training Program, etc.), may have implications for the individual's recidivism and reintegration, providing them with an alternative opportunity for prosocial reintegration into society.

## Data Availability

The original contributions presented in the study are included in the article/supplementary material, further inquiries can be directed to the corresponding author.
