# Supplementary material for: Transcranial magnetic stimulation in forensic populations: scoping review
Source: Front Psychol. 2026 Jun 9;17:1834732. doi: 10.3389/fpsyg.2026.1834732 (PMC13286761; doi:10.3389/fpsyg.2026.1834732)
Supplement: Supplementary file 1 [file Table_1.DOCX]

Supplementary Material

# Appendix A - Appraisal According to STROBE Checklist

**Article:** Hoppenbrouwers et al., 2012

STROBE Statement—checklist of items that should be included in reports of observational studies

|  | Item No. | Recommendation | Page  No. | Relevant text from manuscript | | |
| --- | --- | --- | --- | --- | --- | --- |
| **Title and abstract** | 1 | (*a*) Indicate the study’s design with a commonly used term in the title or the abstract | --- | No information | | |
|  |  | (*b*) Provide in the abstract an informative and balanced summary of what was done and what was found | 1 | e.g., “In the present study we used combined transcranial magnetic stimulation”; “may account for the behavioral impairments associated with this disorder”. | | |
| Introduction | | | | |  |  |
| Background/rationale | 2 | Explain the scientific background and rationale for the investigation being reported | 1-2 | e.g., “Psychopaths are notorious for their callous unemotional personality style”; “both of which are important in the control of behavior”. | | |
| Objectives | 3 | State specific objectives, including any prespecified hypotheses | 2 | e.g., “The aim of this study was”; “We hypothesized that”. | | |
| Methods | | | | |  |  |
| Study design | 4 | Present key elements of study design early in the paper | 2 | e.g., “Thirteen right-handed male psychopathic offenders (…) and fifteen right-handed age-matched healthy male subjects”. | | |
| Setting | 5 | Describe the setting, locations, and relevant dates, including periods of recruitment, exposure, follow-up, and data collection | 2 | e.g., “Psychopathic offenders were recruited through”; “Healthy controls were recruited through”. Sem datas. | | |
| Participants | 6 | (*a*) *Cohort study*—Give the eligibility criteria, and the sources and methods of selection of participants. Describe methods of follow-up  *Case-control study*—Give the eligibility criteria, and the sources and methods of case ascertainment and control selection. Give the rationale for the choice of cases and controls  *Cross-sectional study*—Give the eligibility criteria, and the sources and methods of selection of participants | 2-3 | e.g., “Exclusion criteria included”; “The thirteen psychopathic offenders that were included scored 25 or higher”; “Psychopathology in the control group was ruled out through”. | | |
|  |  | (*b*) *Cohort study*—For matched studies, give matching criteria and number of exposed and unexposed  *Case-control study*—For matched studies, give matching criteria and the number of controls per case | --- | NA | | |
| Variables | 7 | Clearly define all outcomes, exposures, predictors, potential confounders, and effect modifiers. Give diagnostic criteria, if applicable | 3 | e.g., “TMS pulses were administered to”; “In order to exclude a confounding”. | | |
| Data sources/ measurement | 8* | For each variable of interest, give sources of data and details of methods of assessment (measurement). Describe comparability of assessment methods if there is more than one group | 3 | e.g., “CI was indexed through”; “MEPs were measured from”. | | |
| Bias | 9 | Describe any efforts to address potential sources of bias | --- | No information | | |
| Study size | 10 | Explain how the study size was arrived at | --- | No information | | |
| Quantitative variables | 11 | Explain how quantitative variables were handled in the analyses. If applicable, describe which groupings were chosen and why | 4 | e.g., “For every correct sequence one point is given”. | | |
| Statistical methods | 12 | (*a*) Describe all statistical methods, including those used to control for confounding | 4 | e.g., “A multivariate General Linear Model was conducted”; “to exclude a confounding influence of methadone”. | | |
|  |  | (*b*) Describe any methods used to examine subgroups and interactions | --- | No information | | |
|  |  | (*c*) Explain how missing data were addressed | --- | No information | | |
|  |  | (*d*) *Cohort study*—If applicable, explain how loss to follow-up was addressed    *Case-control study*—If applicable, explain how matching of cases and controls was addressed  *Cross-sectional study*—If applicable, describe analytical methods taking account of sampling strategy | --- | NA | | |
|  |  | (*e*) Describe any sensitivity analyses | --- | No information. | | |
| **Results** |  |  |  |  | | |
| Participants | 13* | (a) Report numbers of individuals at each stage of study—eg numbers potentially eligible, examined for eligibility, confirmed eligible, included in the study, completing follow-up, and analysed | 2 | e.g., “Thirty-eight violent offenders were interviewed of which”. Informação no método. | | |
|  |  | (b) Give reasons for non-participation at each stage | 2 | e.g., “although 6 re-offended before they could be included in the study”. | | |
|  |  | (c) Consider use of a flow diagram | --- | No information | | |
| Descriptive data | 14* | (a) Give characteristics of study participants (eg demographic, clinical, social) and information on exposures and potential confounders | 2 | Information in method | | |
|  |  | (b) Indicate number of participants with missing data for each variable of interest | 4 | e.g., “In the control group 10% of all trials were deleted”. | | |
|  |  | (c) *Cohort study*—Summarise follow-up time (eg, average and total amount) | --- | NA | | |
| Outcome data | 15* | *Cohort study*—Report numbers of outcome events or summary measures over time | --- | NA | | |
|  |  | *Case-control study—*Report numbers in each exposure category, or summary measures of exposure | --- | No information | | |
|  |  | *Cross-sectional study—*Report numbers of outcome events or summary measures | --- | NA | | |
| Main results | 16 | (*a*) Give unadjusted estimates and, if applicable, confounder-adjusted estimates and their precision (eg, 95% confidence interval). Make clear which confounders were adjusted for and why they were included | 4-6 | e.g., Figure 1-3; “The post-hoc multivariate GLM that was conducted without the methadone treated subjects to exclude methadone treatment as a potential confound”. | | |
|  |  | (*b*) Report category boundaries when continuous variables were categorized | --- | NA | | |
|  |  | (*c*) If relevant, consider translating estimates of relative risk into absolute risk for a meaningful time period | --- | No information | | |
| Other analyses | 17 | Report other analyses done—eg analyses of subgroups and interactions, and sensitivity analyses | 5 | e.g., “Sham stimulation did not significantly change CI in the M1”. | | |
| **Discussion** |  |  |  |  | | |
| Key results | 18 | Summarise key results with reference to study objectives | 5 | e.g., “we demonstrate that psychopathy is associated with deficient inhibition in the DLPFC”. | | |
| Limitations | 19 | Discuss limitations of the study, taking into account sources of potential bias or imprecision. Discuss both direction and magnitude of any potential bias | 6 | e.g., “Some limitations of the present study should be mentioned”. | | |
| Interpretation | 20 | Give a cautious overall interpretation of results considering objectives, limitations, multiplicity of analyses, results from similar studies, and other relevant evidence | 7 | e.g., “our TMS-EEG study shows”; “Although it is unlikely that deficits in CI and working memory are solely responsible”. | | |
| Generalisability | 21 | Discuss the generalisability (external validity) of the study results | --- | No information | | |
| **Other information** |  |  |  |  | | |
| Funding | 22 | Give the source of funding and the role of the funders for the present study and, if applicable, for the original study on which the present article is based | 7 | e.g., “This work was funded in part by”. | | |

*Note.* *Give information separately for cases and controls in case-control studies and, if applicable, for exposed and unexposed groups in cohort and cross-sectional studies. Adapted from Vandenbroucke et al. (2014, p. 1501).

**Article:** Hoppenbrouwers et al., 2014

STROBE Statement—checklist of items that should be included in reports of observational studies

|  | Item No. | Recommendation | Page  No. | Relevant text from manuscript | | |
| --- | --- | --- | --- | --- | --- | --- |
| **Title and abstract** | 1 | (*a*) Indicate the study’s design with a commonly used term in the title or the abstract | --- | No information | | |
|  |  | (*b*) Provide in the abstract an informative and balanced summary of what was done and what was found | 22 | e.g., “We used transcranial magnetic stimulation combined with”; “Global abnormalities in right to left”. | | |
| Introduction | | | | |  |  |
| Background/rationale | 2 | Explain the scientific background and rationale for the investigation being reported | 22-23 | e.g., “Psychopathic individuals show”; “The combination of TMS and EEG provides a means to”. | | |
| Objectives | 3 | State specific objectives, including any prespecified hypotheses | 23 | e.g., “The aim of the present study was”; “we hypothesized that”. | | |
| Methods | | | | |  |  |
| Study design | 4 | Present key elements of study design early in the paper | 23 | e.g., “Right-handed male psychopathic offenders”; “healthy male control participants”; “6-hour TMS-EEG session and a 2 hour TMS only session”. | | |
| Setting | 5 | Describe the setting, locations, and relevant dates, including periods of recruitment, exposure, follow-up, and data collection | 23 | e.g., “Psychopathic offenders were recruited through”; “a 6-hour TMS-EEG session and a 2 hour TMS only session, which were performed on separate days”. Sem datas. | | |
| Participants | 6 | (*a*) *Cohort study*—Give the eligibility criteria, and the sources and methods of selection of participants. Describe methods of follow-up  *Case-control study*—Give the eligibility criteria, and the sources and methods of case ascertainment and control selection. Give the rationale for the choice of cases and controls  *Cross-sectional study*—Give the eligibility criteria, and the sources and methods of selection of participants | 23 | e.g., “Exclusion criteria for both psychopathic offenders and controls were”; “drug screening”. | | |
|  |  | (*b*) *Cohort study*—For matched studies, give matching criteria and number of exposed and unexposed  *Case-control study*—For matched studies, give matching criteria and the number of controls per case | --- | NA | | |
| Variables | 7 | Clearly define all outcomes, exposures, predictors, potential confounders, and effect modifiers. Give diagnostic criteria, if applicable | 23-25 | e.g., “we measured cortical inhibition and facilitation”; “We measured resting motor threshold”. | | |
| Data sources/ measurement | 8* | For each variable of interest, give sources of data and details of methods of assessment (measurement). Describe comparability of assessment methods if there is more than one group | 24-25 | e.g., “For the motor cortex, the coil was placed over the site that elicited the strongest motor evoked potentials (MEPs) from the abductor pollicis brevis”. | | |
| Bias | 9 | Describe any efforts to address potential sources of bias | --- | No information | | |
| Study size | 10 | Explain how the study size was arrived at | --- | No information | | |
| Quantitative variables | 11 | Explain how quantitative variables were handled in the analyses. If applicable, describe which groupings were chosen and why | --- | NA | | |
| Statistical methods | 12 | (*a*) Describe all statistical methods, including those used to control for confounding | 25 | e.g., “we conducted a repeated measures general linear model”; “A small subgroup (…); therefore, we also conducted analyses excluding these”. | | |
|  |  | (*b*) Describe any methods used to examine subgroups and interactions | --- | No information | | |
|  |  | (*c*) Explain how missing data were addressed | --- | No information | | |
|  |  | (*d*) *Cohort study*—If applicable, explain how loss to follow-up was addressed  *Case-control study*—If applicable, explain how matching of cases and controls was addressed  *Cross-sectional study*—If applicable, describe analytical methods taking account of sampling strategy | --- | NA | | |
|  |  | (*e*) Describe any sensitivity analyses | --- | No information | | |
| **Results** |  |  |  |  | | |
| Participants | 13* | (a) Report numbers of individuals at each stage of study—eg numbers potentially eligible, examined for eligibility, confirmed eligible, included in the study, completing follow-up, and analysed | 25 | e.g., “We enrolled 18 psychopathic offenders”; “Sixteen psychopathic offenders participated in the TMS-EEG session, and 12 participated in the TMS only session. All healthy controls participated in both the TMS-EEG and the TMS only session”. | | |
|  |  | (b) Give reasons for non-participation at each stage | --- | No information | | |
|  |  | (c) Consider use of a flow diagram | --- | No information | | |
| Descriptive data | 14* | (a) Give characteristics of study participants (eg demographic, clinical, social) and information on exposures and potential confounders | 25 | e.g., “mean age”; “The spectrum of criminal activity included”. Informação limitada nesta secção. | | |
|  |  | (b) Indicate number of participants with missing data for each variable of interest | 25 | e.g., “The TMS-EEG ISP data of 1 control participant were excluded (…) and the TMS-EEG ISP data of 1 psychopathic offender were excluded”. | | |
|  |  | (c) *Cohort study*—Summarise follow-up time (eg, average and total amount) | --- | NA | | |
| Outcome data | 15* | *Cohort study*—Report numbers of outcome events or summary measures over time | --- | NA | | |
|  |  | *Case-control study—*Report numbers in each exposure category, or summary measures of exposure | --- | No information | | |
|  |  | *Cross-sectional study—*Report numbers of outcome events or summary measures | --- | NA | | |
| Main results | 16 | (*a*) Give unadjusted estimates and, if applicable, confounder-adjusted estimates and their precision (eg, 95% confidence interval). Make clear which confounders were adjusted for and why they were included | 25-28 | e.g., Table 1; Figure 1-4; “Post hoc repeated-measures general linear models for ISP and CSP were conducted to exclude the potential influence of these confounds”. | | |
|  |  | (*b*) Report category boundaries when continuous variables were categorized | --- | NA | | |
|  |  | (*c*) If relevant, consider translating estimates of relative risk into absolute risk for a meaningful time period | --- | No information | | |
| Other analyses | 17 | Report other analyses done—eg analyses of subgroups and interactions, and sensitivity analyses | --- | No information | | |
| **Discussion** |  |  |  |  | | |
| Key results | 18 | Summarise key results with reference to study objectives | 26-28 | e.g., “found abnormalities in right to left interhemispheric connectivity”. | | |
| Limitations | 19 | Discuss limitations of the study, taking into account sources of potential bias or imprecision. Discuss both direction and magnitude of any potential bias | 29 | e.g., “Several limitations of this study should be mentioned”. | | |
| Interpretation | 20 | Give a cautious overall interpretation of results considering objectives, limitations, multiplicity of analyses, results from similar studies, and other relevant evidence | 29 | e.g., “At present, our data do not provide concrete evidence”. | | |
| Generalisability | 21 | Discuss the generalisability (external validity) of the study results | --- | No information | | |
| **Other information** |  |  |  |  | | |
| Funding | 22 | Give the source of funding and the role of the funders for the present study and, if applicable, for the original study on which the present article is based | 29 | e.g., “Y. Sun declares having received a Master’s Awards from”. | | |

*Note.* *Give information separately for cases and controls in case-control studies and, if applicable, for exposed and unexposed groups in cohort and cross-sectional studies. Adapted from Vandenbroucke et al. (2014, p. 1501).

**Article:** Philipp-Wiegmann et al., 2011

STROBE Statement—checklist of items that should be included in reports of observational studies

|  | Item No. | Recommendation | Page  No. | Relevant text from manuscript | |
| --- | --- | --- | --- | --- | --- |
| **Title and abstract** | 1 | (*a*) Indicate the study’s design with a commonly used term in the title or the abstract | --- | No information | |
|  |  | (*b*) Provide in the abstract an informative and balanced summary of what was done and what was found | 86 | e.g., “we conducted paired-pulse stimulation”; “a reduced cortical inhibition was found”. | |
| Introduction | | | | |  |
| Background/rationale | 2 | Explain the scientific background and rationale for the investigation being reported | 86-87 | e.g., “Aggression is”; “Decreased motor inhibition has been shown”. | |
| Objectives | 3 | State specific objectives, including any prespecified hypotheses | 87 | e.g., “we investigated the primary motor cortex regarding inhibitory control by transcranial magnetic stimulation”; “we hypothesized that”. | |
| Methods | | | | |  |
| Study design | 4 | Present key elements of study design early in the paper | 87 | e.g., “32 were offenders sentenced to prison for violent crimes and 30 were controls”. | |
| Setting | 5 | Describe the setting, locations, and relevant dates, including periods of recruitment, exposure, follow-up, and data collection | 87 | e.g., “The 32 adult male volunteers had been referred to”; “The subjects were assigned to the violent group according to”. Sem datas. | |
| Participants | 6 | (*a*) *Cohort study*—Give the eligibility criteria, and the sources and methods of selection of participants. Describe methods of follow-up  *Case-control study*—Give the eligibility criteria, and the sources and methods of case ascertainment and control selection. Give the rationale for the choice of cases and controls  *Cross-sectional study*—Give the eligibility criteria, and the sources and methods of selection of participants | 87-88 | e.g., “only subjects with habitual aggressive and violent behaviour were included”; “controls had no criminal record”; “Exclusion criteria were”; “drug screening”. | |
|  |  | (*b*) *Cohort study*—For matched studies, give matching criteria and number of exposed and unexposed  *Case-control study*—For matched studies, give matching criteria and the number of controls per case | --- | NA | |
| Variables | 7 | Clearly define all outcomes, exposures, predictors, potential confounders, and effect modifiers. Give diagnostic criteria, if applicable | 88 | e.g., “The resting motor threshold (…) was defined as”; “two magnetic stimuli are delivered”. | |
| Data sources/ measurement | 8* | For each variable of interest, give sources of data and details of methods of assessment (measurement). Describe comparability of assessment methods if there is more than one group | 88 | e.g., “in the resting right-hand first dorsal interosseus muscle”. | |
| Bias | 9 | Describe any efforts to address potential sources of bias | 88 | e.g., “the amplitudes were visually controlled for any abnormalities throughout the measurement”. | |
| Study size | 10 | Explain how the study size was arrived at | --- | No information | |
| Quantitative variables | 11 | Explain how quantitative variables were handled in the analyses. If applicable, describe which groupings were chosen and why | --- | NA | |
| Statistical methods | 12 | (*a*) Describe all statistical methods, including those used to control for confounding | 88 | e.g., “To test the assumption of normal distribution”. | |
|  |  | (*b*) Describe any methods used to examine subgroups and interactions | --- | No information | |
|  |  | (*c*) Explain how missing data were addressed | --- | No information | |
|  |  | (*d*) *Cohort study*—If applicable, explain how loss to follow-up was addressed  *Case-control study*—If applicable, explain how matching of cases and controls was addressed  *Cross-sectional study*—If applicable, describe analytical methods taking account of sampling strategy | --- | NA | |
|  |  | (*e*) Describe any sensitivity analyses | --- | No information | |
| **Results** |  |  |  |  | |
| Participants | 13* | (a) Report numbers of individuals at each stage of study—eg numbers potentially eligible, examined for eligibility, confirmed eligible, included in the study, completing follow-up, and analysed | --- | No information | |
|  |  | (b) Give reasons for non-participation at each stage | --- | No information | |
|  |  | (c) Consider use of a flow diagram | --- | No information | |
| Descriptive data | 14* | (a) Give characteristics of study participants (eg demographic, clinical, social) and information on exposures and potential confounders | 87-88 | Information in method | |
|  |  | (b) Indicate number of participants with missing data for each variable of interest | --- | No information | |
|  |  | (c) *Cohort study*—Summarise follow-up time (eg, average and total amount) | --- | NA | |
| Outcome data | 15* | *Cohort study*—Report numbers of outcome events or summary measures over time | --- | NA | |
|  |  | *Case-control study—*Report numbers in each exposure category, or summary measures of exposure | --- | No information. | |
|  |  | *Cross-sectional study—*Report numbers of outcome events or summary measures | --- | NA | |
| Main results | 16 | (*a*) Give unadjusted estimates and, if applicable, confounder-adjusted estimates and their precision (eg, 95% confidence interval). Make clear which confounders were adjusted for and why they were included | 89-90 | Figure 1-2; Table 1. | |
|  |  | (*b*) Report category boundaries when continuous variables were categorized | --- | NA | |
|  |  | (*c*) If relevant, consider translating estimates of relative risk into absolute risk for a meaningful time period | --- | No information | |
| Other analyses | 17 | Report other analyses done—eg analyses of subgroups and interactions, and sensitivity analyses | 89 | e.g., “In order to control for any effect of severity of violence, we also compared the subgroups of violent offenders”. | |
| **Discussion** |  |  |  |  | |
| Key results | 18 | Summarise key results with reference to study objectives | 90 | e.g., “As expected, no significant difference in RMT was found”. | |
| Limitations | 19 | Discuss limitations of the study, taking into account sources of potential bias or imprecision. Discuss both direction and magnitude of any potential bias | 91 | e.g., “further studies should also investigate violent females”; “a selection bias should be taken into account”. | |
| Interpretation | 20 | Give a cautious overall interpretation of results considering objectives, limitations, multiplicity of analyses, results from similar studies, and other relevant evidence | 90-91 | e.g., “In general, our results suggest that”. | |
| Generalisability | 21 | Discuss the generalisability (external validity) of the study results | --- | No information | |
| **Other information** |  |  |  |  | |
| Funding | 22 | Give the source of funding and the role of the funders for the present study and, if applicable, for the original study on which the present article is based | --- | No information | |

*Note.* *Give information separately for cases and controls in case-control studies and, if applicable, for exposed and unexposed groups in cohort and cross-sectional studies. Adapted from Vandenbroucke et al. (2014, p. 1501).

# Appendix B - Appraisal According to JBI Checklist

**JBI Critical Appraisal Checklist for
case control studies**

Author Hoppenbrouwers et al. Year 2012

|  | Yes | No | Unclear | Not applicable |
| --- | --- | --- | --- | --- |
| 1. Were the groups comparable other than the presence of disease in cases or the absence of disease in controls? | X | □ | □ | □ |
| 1. Were cases and controls matched appropriately? | X | □ | □ | □ |
| 1. Were the same criteria used for identification of cases and controls? | X | □ | □ | □ |
| 1. Was exposure measured in a standard, valid and reliable way? | X | □ | □ | □ |
| 1. Was exposure measured in the same way for cases and controls? | X | □ | □ | □ |
| 1. Were confounding factors identified? | X | □ | □ | □ |
| 1. Were strategies to deal with confounding factors stated? | X | □ | □ | □ |
| 1. Were outcomes assessed in a standard, valid and reliable way for cases and controls? | X | □ | □ | □ |
| 1. Was the exposure period of interest long enough to be meaningful? | X | □ | □ | □ |
| 1. Was appropriate statistical analysis used? | x | □ | □ | □ |

Overall appraisal: Include x Exclude □ Seek further info □

Comments (Including reason for exclusion)

________________________________________________________________________________________________________________________________________________________________________________________________________________________________________________________________________________________________

**JBI Critical Appraisal Checklist for
case control studies**

Author Hoppenbrouwers et al. Year 2014

|  | Yes | No | Unclear | Not applicable |
| --- | --- | --- | --- | --- |
| 1. Were the groups comparable other than the presence of disease in cases or the absence of disease in controls? | X | □ | □ | □ |
| 1. Were cases and controls matched appropriately? | X | □ | □ | □ |
| 1. Were the same criteria used for identification of cases and controls? | X | □ | □ | □ |
| 1. Was exposure measured in a standard, valid and reliable way? | X | □ | □ | □ |
| 1. Was exposure measured in the same way for cases and controls? | X | □ | □ | □ |
| 1. Were confounding factors identified? | X | □ | □ | □ |
| 1. Were strategies to deal with confounding factors stated? | X | □ | □ | □ |
| 1. Were outcomes assessed in a standard, valid and reliable way for cases and controls? | X | □ | □ | □ |
| 1. Was the exposure period of interest long enough to be meaningful? | X | □ | □ | □ |
| 1. Was appropriate statistical analysis used? | x | □ | □ | □ |

Overall appraisal: Include x Exclude □ Seek further info □

Comments (Including reason for exclusion)

________________________________________________________________________________________________________________________________________________________________________________________________________________________________________________________________________________________________

**JBI Critical Appraisal Checklist for
case control studies**

Author Philipp-Wiegmann et al. Year 2011

|  | Yes | No | Unclear | Not applicable |
| --- | --- | --- | --- | --- |
| 1. Were the groups comparable other than the presence of disease in cases or the absence of disease in controls? | X | □ | □ | □ |
| 1. Were cases and controls matched appropriately? | X | □ | □ | □ |
| 1. Were the same criteria used for identification of cases and controls? | X | □ | □ | □ |
| 1. Was exposure measured in a standard, valid and reliable way? | X | □ | □ | □ |
| 1. Was exposure measured in the same way for cases and controls? | X | □ | □ | □ |
| 1. Were confounding factors identified? | X | □ | □ | □ |
| 1. Were strategies to deal with confounding factors stated? | X | □ | □ | □ |
| 1. Were outcomes assessed in a standard, valid and reliable way for cases and controls? | X | □ | □ | □ |
| 1. Was the exposure period of interest long enough to be meaningful? | X | □ | □ | □ |
| 1. Was appropriate statistical analysis used? | x | □ | □ | □ |

Overall appraisal: Include x Exclude □ Seek further info □

Comments (Including reason for exclusion)

________________________________________________________________________________________________________________________________________________________________________________________________________________________________________________________________________________________________
